# A pilot dynamic analysis of formative factors of nephrolithiasis related to metabolic syndrome: evidence in a rat model

**DOI:** 10.1080/0886022X.2022.2097922

**Published:** 2022-07-14

**Authors:** Qiqi He, Yangguo Tang, Yuzhuo Li, Fei Wang, Junsheng Bao, Sanjay Gupta

**Affiliations:** a Department of Urology, Key Laboratory of Disease of Urological Systems, Gansu Nephro-Urological Clinical Center, Lanzhou University Second Hospital, Lanzhou, China; b College of Animal Science and Technology, Guangxi University, Nanning, China; c Department of Paediatric, Lanzhou University Second Hospital, Lanzhou, China; d Department of Urology, University Hospitals Case Medicine Center, Case Western Reserve University, Cleveland, OH, USA

**Keywords:** Nephrolithiasis, metabolic syndrome, high oxalate urine, animal model

## Abstract

**Introduction and objective:**

To examine the dynamic changes in the formative factors of nephrolithiasis and the final micromorphological changes in an obesity-initiated metabolic syndrome (MS) rat model.

**Methods:**

Forty five-week-old male Sprague–Dawley (SD) rats were randomly divided into four groups: the regular diet group (RD), high-fat diet group (HFD), regular diet with drug (ethylene glycol and ammonium chloride) group (RDD), and high-fat diet with drug group (HFDD). A dynamic assessment of MS components (body weight (BW), body length (BL), Lee’s index (LI), blood glucose (BG), total cholesterol (TC), and triglycerides (TGs)) and stone-forming factors (urinary pH, urinary calcium, and urinary oxalate acid) was carried out. In addition, the levels of oxidative stress (OS) markers (CAT, SOD, TAC, GSH-PX, and MDA) were measured, and histological analysis was carried out at the end of 16 weeks.

**Results:**

MS-related parameters, such as BW, LI, BG, TC, and TG, were significantly higher in HFD-fed rats than in RD-fed rats (*p* < 0.001). In the HFDD group, significantly lower urinary pH, hyperoxaluria, and hypocalciuria were noted in the dynamic assessment of stone-forming factors (*p* < 0.001). CAT, TAC, and MDA were notably changed in the HFD-fed groups, particularly the HFDD rats. Histological analysis showed that the renal tubules of HFDD rats had the highest scores for both inflammation and renal crystallization deposition (*p* < 0.05).

**Conclusions:**

Our results suggest that male SD rats with MS are prone to developing nephrolithiasis. Validation in an *in vivo* model may lead to an understanding of the underlying pathophysiological mechanisms of action of MS-related nephrolithiasis in humans.

**Key messages**
- Male SD rats with metabolic syndrome are more prone to developing calcium oxalate nephrolithiasis after treatment with ethylene glycol and ammonium chloride compared to control lean rats.
- MS-related nephrolithiasis in rats induced by ethylene glycol and ammonium chloride is mainly related to increased hyperoxaluria and inflammation and decreased antioxidant levels.
- High-fat diet-fed SD rats treated with ethylene glycol and ammonium chloride are a stable and valid *in vivo* model for understanding the potential mechanism of action of MS-related nephrolithiasis.

## Background

Nephrolithiasis is one of the most common urological diseases and has high morbidity and recurrence rates. This disease has been documented to affect approximately 5–10% of individuals, and the recurrence rate within 5 years could be as high as 50–70% worldwide [1,2]. Metabolic syndrome (MS) was proposed as an umbrella term for cardiovascular and metabolic risk factors, including obesity, hypertension, hyperglycemia, and hypertriglyceridemia, that can predict cardiovascular-metabolic complications and even death. The prevalence of MS affects approximately 34–39% of the adult population worldwide [3,4].

Numerous clinical studies have demonstrated that the risk of nephrolithiasis is closely related to MS and its components [5–8]. Approximately 48.7% of nephrolithiasis patients in the United States have MS characteristics. Patients with more than three MS components have a significantly increased incidence of stone diseases, and patients with four MS components have a 1.8 times higher risk of nephrolithiasis than individuals in the normal control group [8]. One recent systematic review that included 25 potentially relevant studies (*n* = 934,588 participants) suggested that all components of MS were linked to an increased incidence of nephrolithiasis. The possible mechanisms associated with MS are often related to lipotoxicity and insulin resistance, which induce systemic inflammation and oxidative stress [9]. However, this link has been explored for only a short time, and there are clinical limitations. Relatively little evidence from animal models has been published on the study of the underlying mechanisms of the link between MS and nephrolithiasis. To our knowledge, only one study showed that proinflammatory adipocytokines and macrophages facilitate renal crystal formation in ob/ob mice with MS(17).

This study was designed to examine the dynamic characteristics of the determining factors of nephrolithiasis, examine the final micromorphological manifestations and identify potential pathomechanisms in a rat model of MS. Overall, our data address the question of whether high-fat diet-fed rats can serve as a suitable translational model to study the link between MS and nephrolithiasis. Moreover, we demonstrate that Sprague–Dawley (SD) rats fed a high-fat diet (HFD) are prone to the development of nephrolithiasis mainly via the mediation of inflammatory injury in nephron tubules caused by oxidative stress and hyperoxaluria.

## Materials and methods

### Animals

**Figure 1.**
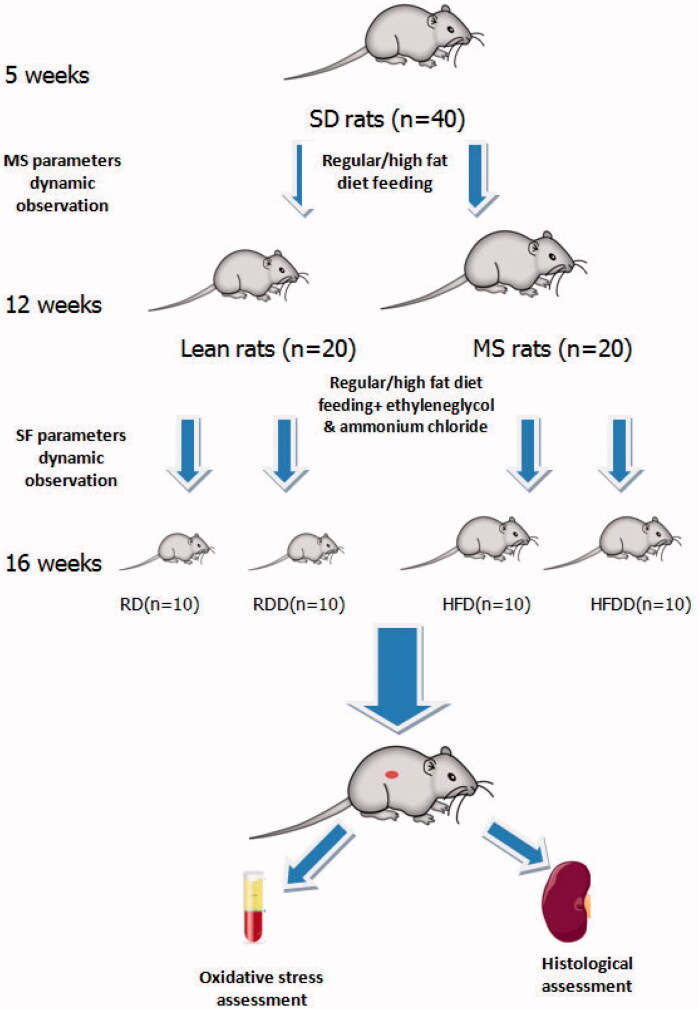
Study design and grouping description. (MS: Metabolic syndrome, SF: Stone-forming, RD: Regular diet + saline, RDD: Regular diet + ethylene glycol and ammonium chloride, HFD: High fat diet + saline, HFDD: High fat diet + ethylene glycol and ammonium chloride)

Forty five-week-old male SD rats (body weight: 146–160 g, body length: 17–18 cm) were purchased from Guangxi Medical University and housed at 22–26 °C under a 12:12 h light–dark cycle (lights on from 7:00 AM to 7:00 PM). All rats were randomly divided into four groups and marked with different colors for identification. They were given access to different rodent diets and drug treatments. Two groups of rats (*n* = 20) were fed a HFD (20% protein + 20% carbohydrate + 60% fat) to establish the MS rat model, and the other two groups (*n* = 20) were fed a regular diet (20% protein + 76% carbohydrate + 4% fat) to serve as the lean control groups. All rats were maintained on their diets for 8 weeks. At 12 weeks of age, all rats were further grouped (*n* = 10) according to drug treatment (1% ethylene glycol, po + 2% ammonium chloride, ip) or blank placebo (saline, po + ip) by the sortition randomization method until 16 weeks of age (Figure 1). All experimental protocols were approved by the Institutional Animal Care and Use Committee and ethics committee for animal research of Lanzhou University Second Hospital (2021 A-006), which strictly followed and was in accordance with the international regulations for lab animal care and use.

### Serum and urine assays

#### Parameters of metabolic syndrome

Body weight (BW) and body length (BL, nose to anus) were measured once a week. Lee's index (LI) was calculated by the following formula: Lee's index = body weight (g)^1/3^ ×10^3^/body length (cm). All blood samples were collected from the tail vein. Fasting blood glucose (BG) was measured with an Accucheck1 Aviva glucose meter (Roche Diagnostics, Indianapolis, IN, USA) every 2 weeks. At the end of 12 weeks (8 weeks of MS modeling), plasma triglycerides (TGs) were measured using a rat/mouse ELISA kit (Nanjing Jiancheng Technology, China), and total cholesterol (TC) levels were measured using a cholesterol assay kit (Nanjing Jiancheng Technology, China).

#### Parameters of nephrolithiasis formation

After MS modeling was successfully established at 12 weeks, drug administration was carried out to induce nephrolithiasis in rats. The urine samples of rats were collected in metabolic cages to perform a routine urine test (URITEST-500B urine analyzer, Guangxi, China) and measure urinary oxalate (Oxalate Assay kit, Nanjing Jiancheng Technology, China) and urinary calcium (calcium assay kit, Nanjing Jiancheng Technology, China) levels once a week.

#### Parameters of oxidative stress

The rats were euthanized by overdose of barbiturates at the end of 16 weeks. Blood was obtained from the aorta to assess oxidative stress markers, including catalase (CAT), total antioxidative capacity (TAC), glutathione peroxidase (GSH-PX), superoxide dismutase (SOD) and malondialdehyde (MDA) (oxidative stress assay kit, Nanjing Jiancheng Technology, China).

### Histological characterization of inflammation and crystal deposition

Renal tissue samples were obtained, weighed and then fixed in 4% paraformaldehyde for routine histological examination. Paraffin-embedded renal sections were cut and stained. Each slide was stained with hematoxylin and eosin (HE) and used to calculate the volume of inflammation based on counts of infiltrating inflammatory cells (% in total tubules) by two observers who were blinded to the group assignments. Moreover, Pizzolato staining was performed on other slides to count crystal deposits (% in total tubules) at 100× magnification [10].

### Statistical analysis

Data were analyzed using the SPSS 22.0 software package (IBM SPSS Statistics, NY, US). Numerical variables that were normally distributed are expressed as the mean ± SEM and were analyzed by independent sample *t* test. Four groups of animals were compared using the chi-square test for categorical variables and ANOVA or the Kruskal–Wallis test for quantitative variables. All statistical tests of hypotheses were two-sided, and *p* values less than 0.05 were considered indicative of statistical significance.

## Results

**Figure 2.**
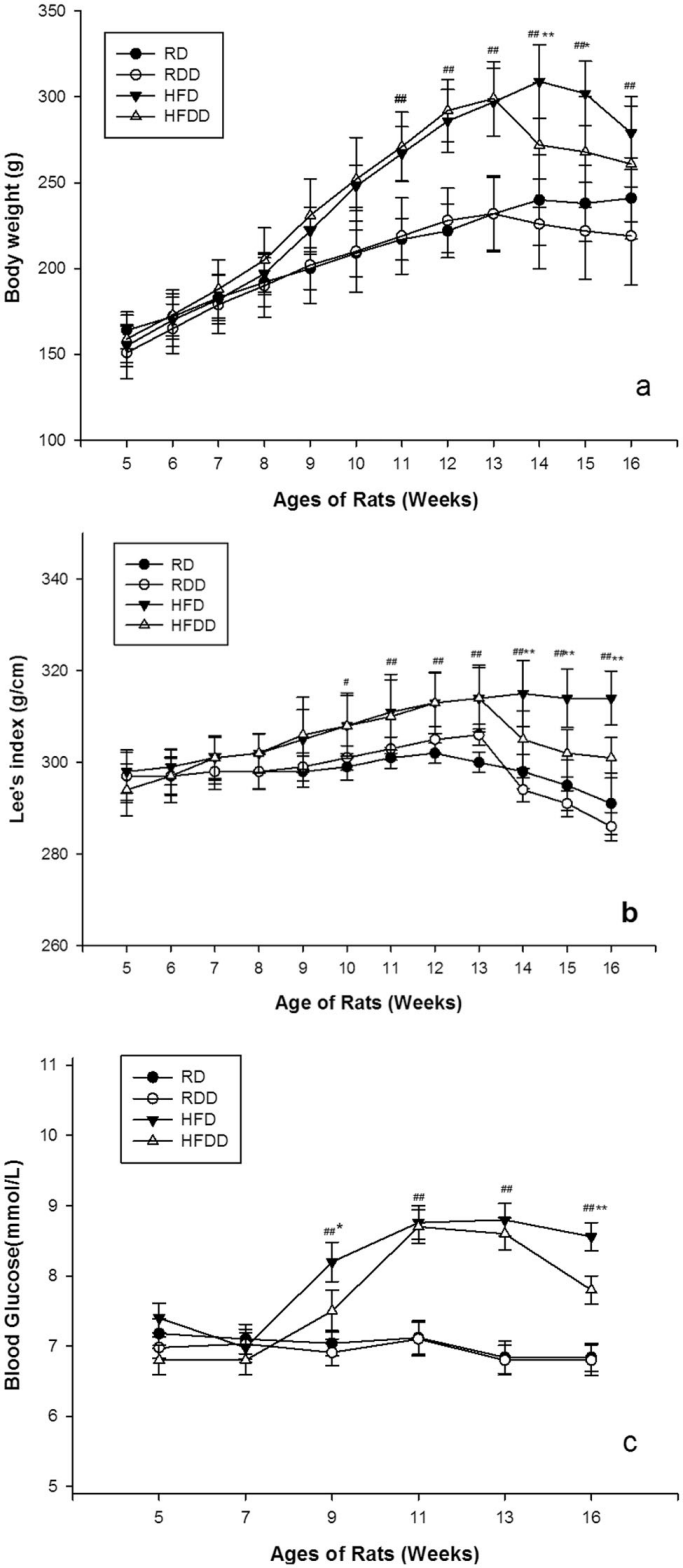
Dynamic assessment of obesity parameters during MS modeling: body weight (a), Lee’s index (b), and fasting blood glucose (c) (#*p* < 0.05, ##*p* < 0.01 vs. RD, *p < 0.05, **p < 0.01 vs. other 3 groups). Details are described in ‘Materials and Methods’ section.

The characteristics of the MS and control lean rats at 12 weeks were assessed and are shown in Table 1. Compared to the control groups, the MS groups showed statistically significant increases in BW (*p* < 0.001) and LI (*p* < 0.001) at each time point (Figure 2). No significant differences were noted in body length (data not shown). In terms of metabolic markers of MS, the BG level in the MS group was significantly increased at 12 weeks (Figure 2). We also confirmed the levels of TG (*p* < 0.001) and TC (CHO) (*p* < 0.001) increased by almost 200% throughout the study in MS rats compared to the control lean rats (Table 1). These results confirm that HFD-fed rats exhibit components of MS, including increased BMI, higher fasting BG levels, hypercholesterolemia and hyperlipidemia at 12 weeks.

**Table 1.** The characteristics of MS/Control group induced by different diet at 12 weeks.

Characteristics	Control (*n* = 20)	MS (*n* = 20)	*p* Value
Body weight (g)	225.30 ± 5.19	289.50 ± 25.61	**<0.001**
Body length (cm)	20.00 ± 0.30	21.10 ± 0.74	0.725
Lee’s index (g/cm)	304.42 ± 2.34	313.45 ± 9.08	**<0.001**
Blood glucose (mmol/L)	7.12 ± 0.16	7.75 ± 0.41	**<0.001**
Total cholesterol (mmol/L)	2.99 ± 0.85	4.06 ± 1.09	**<0.001**
Triglyceride (mmol/L)	0.70 ± 0.35	1.52 ± 0.41	**<0.001**

*p* values in bold mean significant differences.

Values represent Mean ± SEM, Details are described in ‘Materials and Methods’ section.

**Figure 3.**
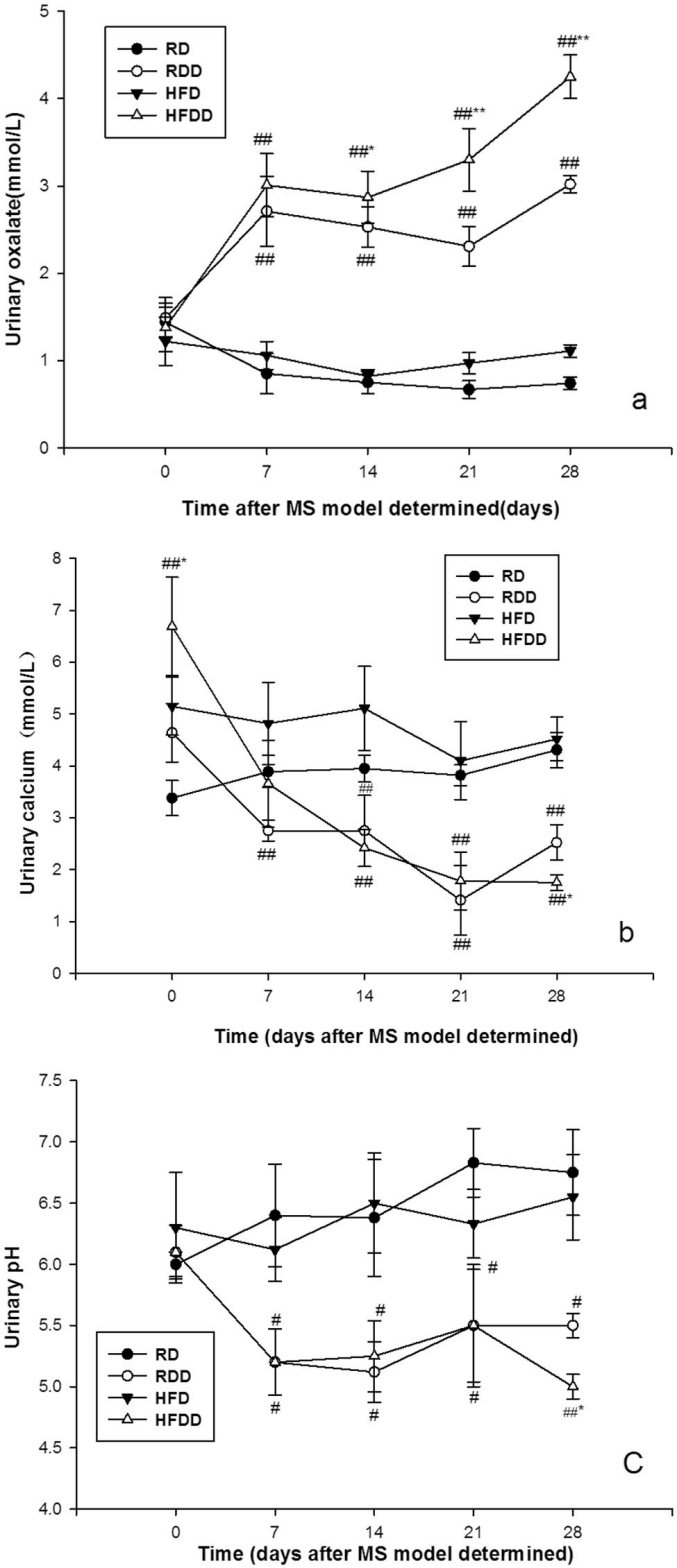
Dynamic assessment of renal stone-forming factors after MS modeling: urinary oxalate (a), urinary calcium (b), and urinary pH (c) (#*p* < 0.05, ##*p* < 0.01 vs. RD, *p < 0.05, **p < 0.01 vs other 3 groups).Details are described in ‘Materials and Methods’ section.

Next, we measured the dynamic changes in the MS characteristics and stone-forming factors in these rats. The characteristics of the four groups at 16 weeks are shown in Table 2. The HFD group showed a statistically significant increase in all MS components (*p* < 0.001). In terms of stone-forming factors, the HFDD group showed a statistically significant increase in urine oxalate and a decrease in urine calcium at most time points compared to the other groups (*p* < 0.001). The urine pH in this group at 16 weeks was significantly lower than that of the other three groups (*p* < 0.001) (Figure 3 and Table 2).

**Table 2.** The characteristics of four groups at the end of 16 weeks.

Characteristics	RD (*n* = 10)	RDD (*n* = 10)	HFD (*n* = 10)	HFDD (*n* = 10)	*p* Value
MS parameters					
Body weight (g)	241.80 ± 10.20	218.80 ± 12.91	297.00 ± 23.61	261.80 ± 9.28	**<0.001**
Body length (cm)	21.40 ± 0.41	21.00 ± 0.54	21.30 ± 0.71	21.70 ± 0.44	0.798
Lee’s index (g/cm)	291.16 ± 6.68	286.99 ± 6.65	314.67 ± 5.91	301.78 ± 4.41	**<0.001**
Blood glucose (mmol/L)	6.84 ± 0.45	6.76 ± 0.54	8.86 ± 0.32	7.86 ± 0.39	**<0.001**
Total cholesterol (mmol/L)	2.90 ± 0.59	3.00 ± 0.63	4.20 ± 0.92	4.33 ± 0.65	**<0.001**
Triglyceride (mmol/L)	0.60 ± 0.22	0.68 ± 0.37	1.31 ± 0.16	1.46 ± 0.20	**<0.001**
Stone former parameters					
Urinary PH	6.75 ± 0.35	5.50 ± 0.35	6.75 ± 0.35	5.00 ± 0.50	**<0.001**
Oxaluria (mmol/L)	0.74 ± 0.07	3.02 ± 0.10	1.11 ± 0.07	4.25 ± 0.25	**<0.001**
Calciuria (mmol/L)	4.31 ± 0.34	2.52 ± 0.34	4.54 ± 0.42	1.75 ± 0.05	**<0.001**
Serum calcium (mmol/L)	1.38 ± 0.22	1.35 ± 0.04	1.35 ± 0.06	1.32 ± 0.04	0.685
Oxidative stress status					
CAT (U/ml)	4.33 ± 1.81	2.75 ± 1.54	3.69 ± 2.33	1.83 ± 1.42	**<0.001**
TAC (mM)	0.41 ± 0.06	0.34 ± 0.09	0.35 ± 0.07	0.25 ± 0.02	**0.017**
SOD ( U/ml)	591.71 ± 32.17	576.67 ± 38.34	585.49 ± 20.69	566.72 ± 32.83	0.631
GSH-PX (U)	1304.09 ± 325.37	1268.74 ± 549.41	1114.23 ± 172.79	1281.83 ± 394.71	0.690
MDA (nmol/mL)	11.67 ± 2.20	10.68 ± 1.96	15.23 ± 0.56	14.91 ± 1.74	**0.001**
Histological Characteristics					
Renal weight (mg)	943.80 ± 100.31	1011.77 ± 44.65	981.37 ± 99.80	1384.00 ± 272.34	**0.003**
Inflammation count (% tubules, 100×)	4.48 ± 0.06	22.19 ± 0.08	13.01 ± 0.07	69.60 ± 0.14	**<0.001**
Crystal deposits count (% in tubules, 100×)	0	2.30 ± 0.51	0	18.82 ± 5.28	**<0.001**

These data compared using the chi-square test for categorical variables and ANOVA or the Kruskal–Wallis test for quantitative variables, *p*-value in bold means statistic significant. Details are described in ‘Materials and Methods’ section.

**Figure 4.**
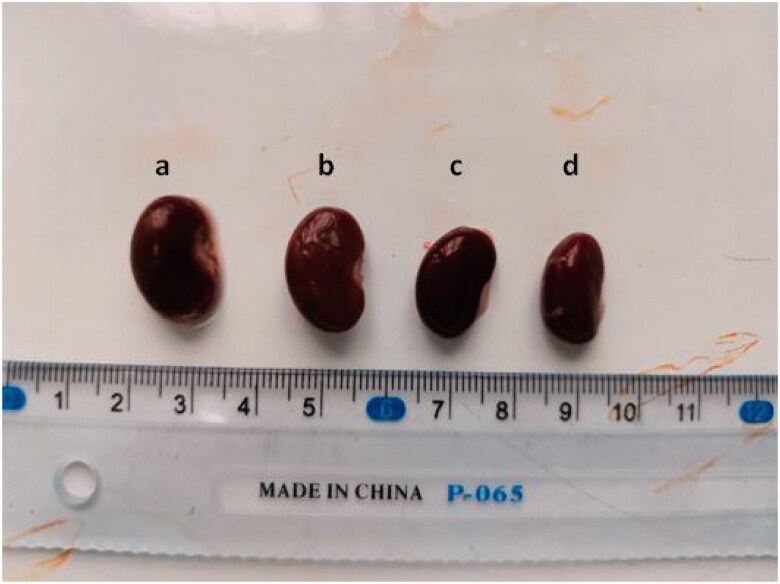
The representative morphology of kidney specimens in the four groups at 16 weeks. (a. HFDD, b. HFD, c. RDD, d. RD)

At the end of 16 weeks, all rats were sacrificed, renal specimens were harvested, and blood was collected from the aorta for further assays. Gross observation of renal specimens indicated that compared with the other groups, the HFDD group had larger kidneys with a dark brown cortex and granules (Figure 4). The renal weight in the HFDD group was significantly higher than that of the other groups (*p* < 0.001) (Table 2).

Next, we compared the oxidative stress levels among the four groups. The levels of CAT and TAC were markedly decreased in the HFDD group compared with the other groups (*p* < 0.001), whereas MDA was increased in both the HFD and HFDD groups (*p* < 0.001). No significant differences were noted in SOD or GSH-px (Table 2).

**Figure 5.**
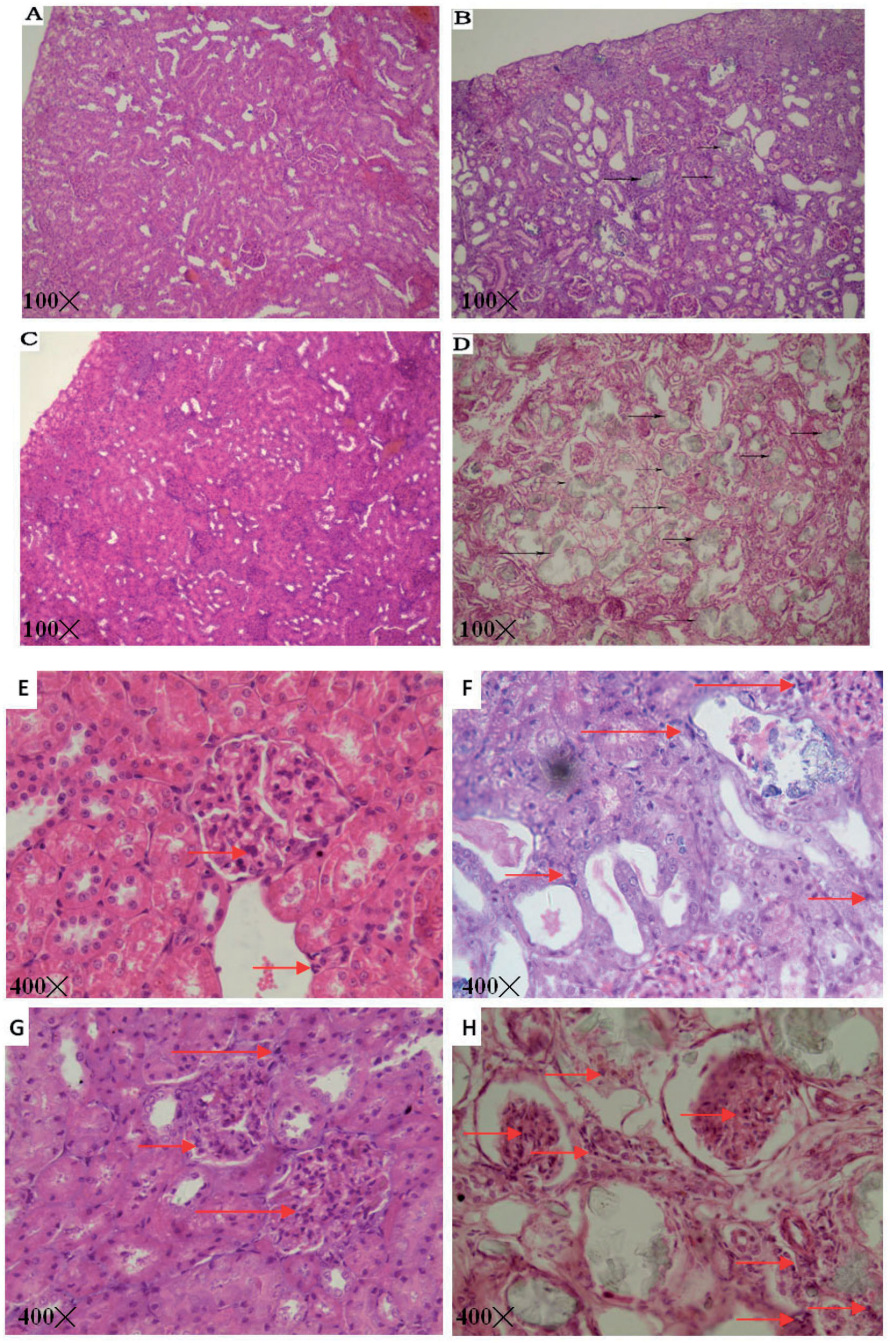
Representative image of H&E staining of renal tissue (100× and 400× magnification; A&E: RD, B&F: RDD, C&G: HFD, D&H: HFDD; black arrow: crystal deposition, red arrow: inflammatory cells).

**Figure 6.**
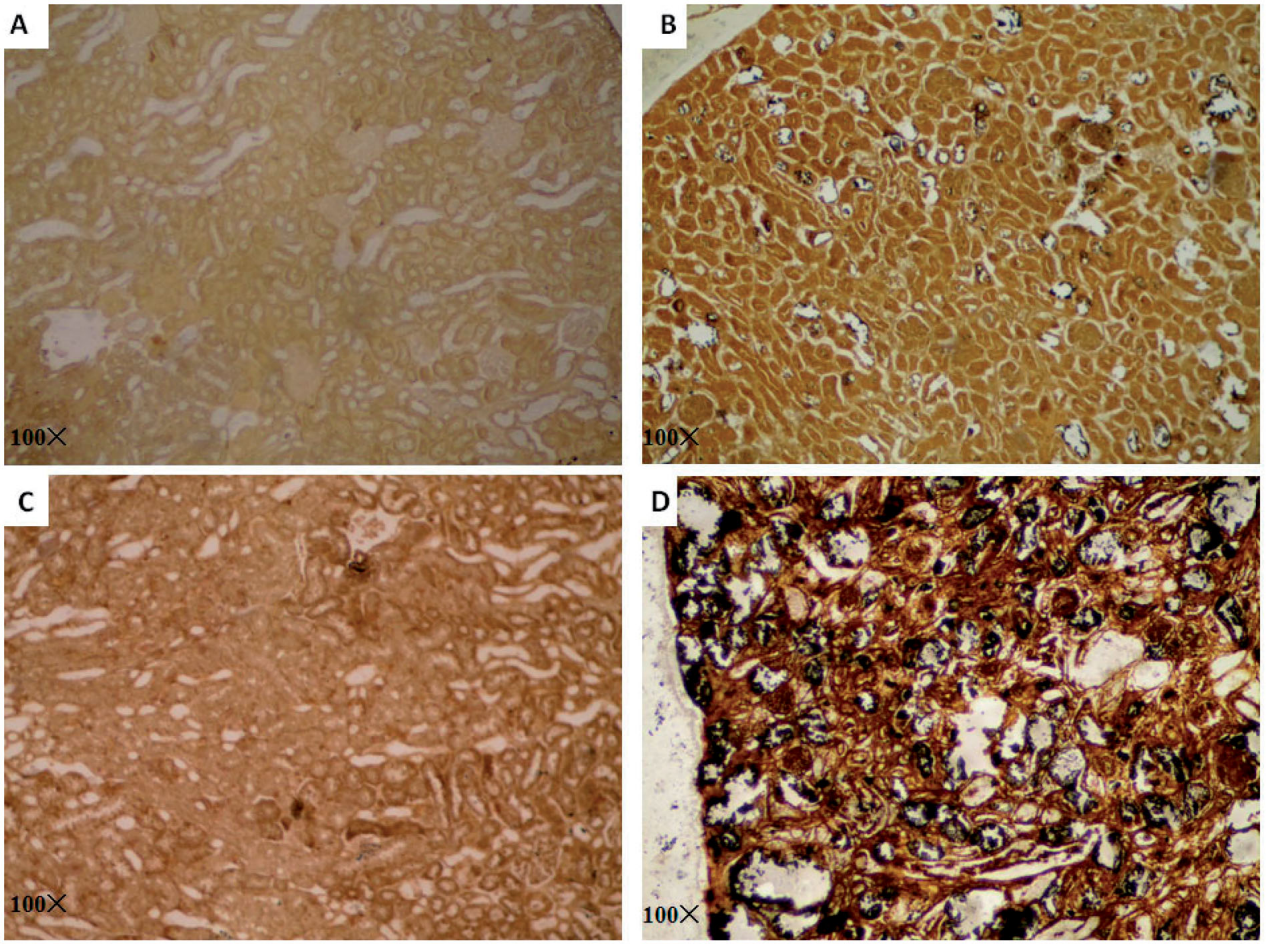
Representative images of Pizzolatto staining of renal tissue (100× magnification) (A: RD, B: RDD, C: HFD, D: HFDD).

Histological examination of the renal tissue of the four groups showed pronounced infiltration of inflammatory cells (*p* < 0.001) and crystal deposition (*p* < 0.001) in the HFDD group (Figure 5 and Table 2). These results indicated that in rats, MS increases inflammation and promotes crystal deposition compared to the control condition. The structure of the nephron was markedly loose and extensively damaged, virtually filling the deformed lumen with transparent crystals and resulting in severe inflammation in the HFDD group. The aggregates were qualitatively categorized as infiltrating immune cells and were markedly more numerous in the nephrons of HFDD rats compared to those of the other groups (*p* < 0.001) under 100 and 400× magnification (Figure 5). Pizzolatto staining stains oxalate calcium (CaOX) stones black in tissue, which allows for the qualitative and quantitative identification of CaOX in renal tissue. Large amounts of black CaOX crystals were observed in the renal tubules of the HFDD group, while small amounts were observed in the tubules of the RDD group (*p* < 0.001). No significant black staining was observed in the RD or HFD group (Figure 6 and Table 2).

## Discussion

Our main purpose in this study was to investigate the dynamic changes in the formative factors of nephrolithiasis in an MS rat model. The results indicated that male SD rats with MS are more prone to developing calcium oxalate nephrolithiasis after treatment with ethylene glycol and ammonium chloride than control lean rats. The mechanism of action is mainly related to increased hyperoxaluria and inflammation and decreased antioxidant levels. We also confirmed that high-fat diet-fed SD rats treated with ethylene glycol and ammonium chloride are a stable and valid model *in vivo* for understanding the potential mechanisms of MS-related nephrolithiasis.

The main pathological components of MS include obesity, insulin resistance, hypertension and dyslipidaemia, and MS is becoming one of the great challenges in public health worldwide [4]. The relationship between MS and nephrolithiasis has been well established by many clinical trials. Many studies indicate a close link between obesity and an increased risk of nephrolithiasis. Taylor and colleagues utilized three epidemiological prospective cohort studies with 4827 cases to identify that nephrolithiasis was directly correlated with weight and high BMI (>30 kg/m^2^). Increased visceral adipose tissue was reported to be associated with the risk of developing CaOx and uric acid stones [11]. Similarly, other studies have demonstrated the links between the risk of nephrolithiasis and insulin resistance [12], hypertension [13], dyslipidaemia [14], cardiovascular disease (CVD) and some other MS components [15], obtaining compelling positive evidence. However, as multiple confounding factors exist in MS, the specific pathophysiological molecular mechanisms underlying the link between MS and nephrolithiasis remain unclear.

Animal models play a critical role in mechanistic studies of disease. Furthermore, animal models of MS have been widely used in CVD, type 2 diabetes, nonalcoholic fatty liver disease, malignant tumors (liver, pancreas, breast and bladder), and kidney and pancreatic dysfunction since 2005 [16]. However, MS-related nephrolithiasis has not drawn much attention until recent years. Although many epidemiological surveys and clinical trials have indicated that a strong link exists between MS and nephrolithiasis, a lack of evidence from animal models for this topic remains. Taguchi K et al. induced renal crystal deposition in ob/ob mice with MS using a high-fat diet and ethylene glycol, which suggests that proinflammatory adipocytokines and macrophages facilitate renal crystal formation in mice [17]. Javier Sáenz-Medina et al. investigated the influence of MS on nephrolithiasis using a murine model. The results indicated that in an MS rat model, hyperoxaluria might cause severe morphological alterations with a significant impairment of renal function because of enhanced inflammation. However, the relationship between inflammation and potential oxidative stress was not analyzed. Moreover, the 65 days of treatment with a 60% fructose diet in their study did not successfully induce MS features other than dyslipidaemia, and thus, their model should not be recognized as a successful MS rat model [18]. Our previous studies [19,20] also indicated that MS could be induced in ob/ob mice fed normal chow at 28 weeks of age. However, renal crystal deposition might not be easily observed in mice without drug induction, and the classic method of crystal induction by ethylene glycol injection might pose a life-threatening risk to ob/ob mice according to our experiences. Due to these limitations in the literature, we reasoned that conducting this study to define the characteristics of MS-related nephrolithiasis, including oxidative stress and micromorphological manifestations, would be meaningful.

The highlight of the current study, is that it is, to the best of our knowledge, the first study to continuously and dynamically explore the development of formative factors of nephrolithiasis in a rat model that could perfectly mimic most features of MS, such as body weight gain, larger waist circumference, hyperglycemia, hypercholesterolemia, and hyperlipidemia. This study also finally defined the micromorphological characteristics of MS-induced nephrolithiasis. In our study, the pathogenesis of MS-initiated nephrolithiasis might be primarily related to hyperoxaluria, hypocalciuria and lower urine pH based on our dynamic assessment.

Animals with MS were more prone to developing hyperoxaluria, which has been widely reported [17,21]. Taguchi K et al. carefully dissected the potential underlying mechanisms of obesity-associated hyperoxaluria in ob/ob mice. However, excluding the contribution of dietary indiscretion, urinary oxalate remained significantly higher in ob/ob mice, suggesting that hyperoxaluria was not solely due to increased oxalate consumption. Hyperoxaluria in MS might be related to systemic inflammation. In particular, an increase in proinflammatory cytokine levels in the intestine could suppress oxalate secretion and contribute to hyperoxaluria [17]. In our study, we found more significant persistent hyperoxaluria in the HFDD group than in the RDD group at each time point. This result indicates that MS promotes the development of hyperoxaluria, which generates inflammation and OS in renal tissue to accelerate crystal formation and deposition.

Hypocalciuria was notably observed in the MS rats in our study, which is not consistent with the general risk factors theory of hypercalciuria in the regular stone pathogenic mechanism. To our knowledge, no literature has demonstrated and explained this manifestation and general mechanism. Only one clinical study has shown that urinary calcium has a very weak correlation with components of MS [22]. Due to limited literature, we hypothesize that the hypocalciuria in our study might be related to large amounts of ROS released in tissue. We also hypothesize that the long-term effects of excessive ROS would cause lipid peroxidation on the cell membrane and increase the permeability of the cell membrane and influx of calcium ions (Ca^2+^). All these circumstances induce continuous depolarization of mitochondria, inhibition of ATP production, and overload of intracellular Ca^2+^. Continuous Ca^2+^ influx might cause hypocalciuria, mitochondrial fragmentation, and inflammatory responses. Consistently, one recent study [23] indicated that mitochondrial dysfunction and fragmentation induced by excessive reactive oxygen species (ROS) in MS might promote calcium deposition on the surface of renal tubular epithelial cells and aggregation into crystals. Hypercalciuria is usually secondary to renal tubular acidosis and hyperparathyroidism. It leads to disturbances in calcium homeostasis and is recognized as a risk factor for nephrolithiasis. This finding might be more accurate and reliable with an increase in sample size. Due to ammonium chloride administration, lower urine pH levels were observed in both drug administration groups.

In addition, we explored the parameters of oxidative stress and histological changes in MS-related nephrolithiasis. Oxidative stress is well known to play an important role in both MS and nephrolithiasis. The results of our study indicated a poor antioxidative capacity in rats in the HFDD group, which are more sensitive to inflammation. The oxidative stress in HFDD rats is derived from both high-fat diet feeding and drug administration. Both interventions increase ROS production, which can cause mtDNA damage followed by alterations in the mitochondrial fission/fusion process. These alterations can lead to cellular injury, apoptosis, the inflammatory response, and finally disease progression. Excessive ROS and inflammatory cytokine levels could decrease CAT and TAC levels. MDA is a marker of cell membrane lipid peroxidation related to membrane permeability and was elevated in both the high-fat diet-fed groups. Membrane permeability usually causes intracellular calcium levels to change. Calcium overload may cause mitochondrial dysfunction and ROS overproduction, which aggravate OS and the inflammatory response. Several studies [10,24,25] have shown that the development of renal tubular injury is related to the excessive ROS generated by oxalate, which is involved in NF-κB/TGF-β activation. Further mechanistic studies on mitochondrial functions, OS, and inflammation-specific pathways and responses in MS-related nephrolithiasis are needed.

The histopathological examination in our study demonstrated massive destruction and dilation of tubules and intraluminal crystal aggregates in the HFDD group. These findings could explain the observed differences in renal weights and gross manifestations, which indicated that SD rats with MS are prone to developing renal inflammation and nephrolithiasis. Polymorphonuclear cells could also be observed within the renal tubules, which is a sign of inflammation. Moreover, to our knowledge, our study is the first to demonstrate crystal deposition by Pizzolatto staining of renal tubules in an MS animal model. Sharp contrast staining leads to a higher sensitivity and specificity in calculating the CaOX deposition proportion in the tubules, which might be beneficial to improving our understanding of MS-related nephrolithiasis.

However, similar to any animal model studies exploring the potential mechanism of diseases, several limitations in this study need to be taken into account. One limitation is that the sample sizes and parameters detected were limited, and thus, these results cannot reflect the comprehensive manifestations of formative factors of MS-related nephrolithiasis in the clinic. Some formative factors, such as urine citrate level and ureteral malformation. were overlooked. Moreover, rodent gut microbiota and metabolic capacity are significantly different from those of humans, and MS-related nephrolithiasis in humans is not induced by ethylene glycol or ammonium chloride. The pathogenic differences among species exist and cannot be overlooked. Whether this animal model is appropriate for exploring the possible molecular mechanism of MS-related nephrolithiasis requires further study.

## Conclusions

In summary, we dynamically illustrate the main formative characteristics in an effective animal model that mimics the features of MS and nephrolithiasis. This model recapitulates the findings of several epidemiologic and case–control studies of human populations that are associated MS with nephrolithiasis. Our study suggests that MS could be induced in SD rats via high-fat diet feeding, which might increase the likelihood of the development of calcium oxalate nephrolithiasis. The pathogenesis may be primarily related to hyperoxaluria, inflammation, and low antioxidant levels. Further investigations are needed to confirm these findings.

## Data Availability

All data, models, and code generated or used during this study appear in the article.

## Abbreviations

MS: metabolic syndrome
BW: body weight
BL: body length
LI: Lee’s index
BG: blood glucose
TC: total cholesterol
TG: triglyceride
OS: oxidative stress
CAT: catalase
TAC: total antioxidative capacity
GSH-PX: glutathione peroxidase
SOD: superoxide dismutase
MDA: malondialdehyde
po: per os
ip: intraperitoneal injection
